# 3,6-Di­chloro-9-(prop-2-yn-1-yl)-9*H*-carbazole

**DOI:** 10.1107/S1600536813032777

**Published:** 2013-12-07

**Authors:** Joel T. Mague, Mehmet Akkurt, Shaaban K. Mohamed, Asmaa H. Mohamed, Mustafa R. Albayati

**Affiliations:** a Department of Chemistry, Tulane University, New Orleans, LA 70118, USA; b Department of Physics, Faculty of Sciences, Erciyes University, 38039 Kayseri, Turkey; c Chemistry and Environmental Division, Manchester Metropolitan University, Manchester M1 5GD, England; d Chemistry Department, Faculty of Science, Minia University, 61519 El-Minia, Egypt; e Kirkuk University, College of Science, Department of Chemistry, Kirkuk, Iraq

## Abstract

The tricyclic aromatic ring system of the title compound, C_15_H_9_Cl_2_N, is essentially planar (r.m.s. deviation = 0.002 Å). The two Cl atoms lie slightly out of the plane of the carbazole ring system, with the C—Cl bonds forming angles of 1.23 (8) and 1.14 (8)° with the plane. The acetylene group has a *syn* orientation with respect to the ring system. In the crystal, no weak hydrogen bonds nor any π–π stacking inter­actions are observed.

## Related literature   

For industrial applications of carbazole-containing compounds, see: Zhang *et al.* (1998 ▶). For pharmaceutical properties of carbazoles, see: Liu & Larock (2007 ▶); Hussain *et al.* (2011 ▶); Zhang *et al.* (2010 ▶); Conchon *et al.* (2006 ▶). For a related structure, see: Xie *et al.* (2012 ▶).
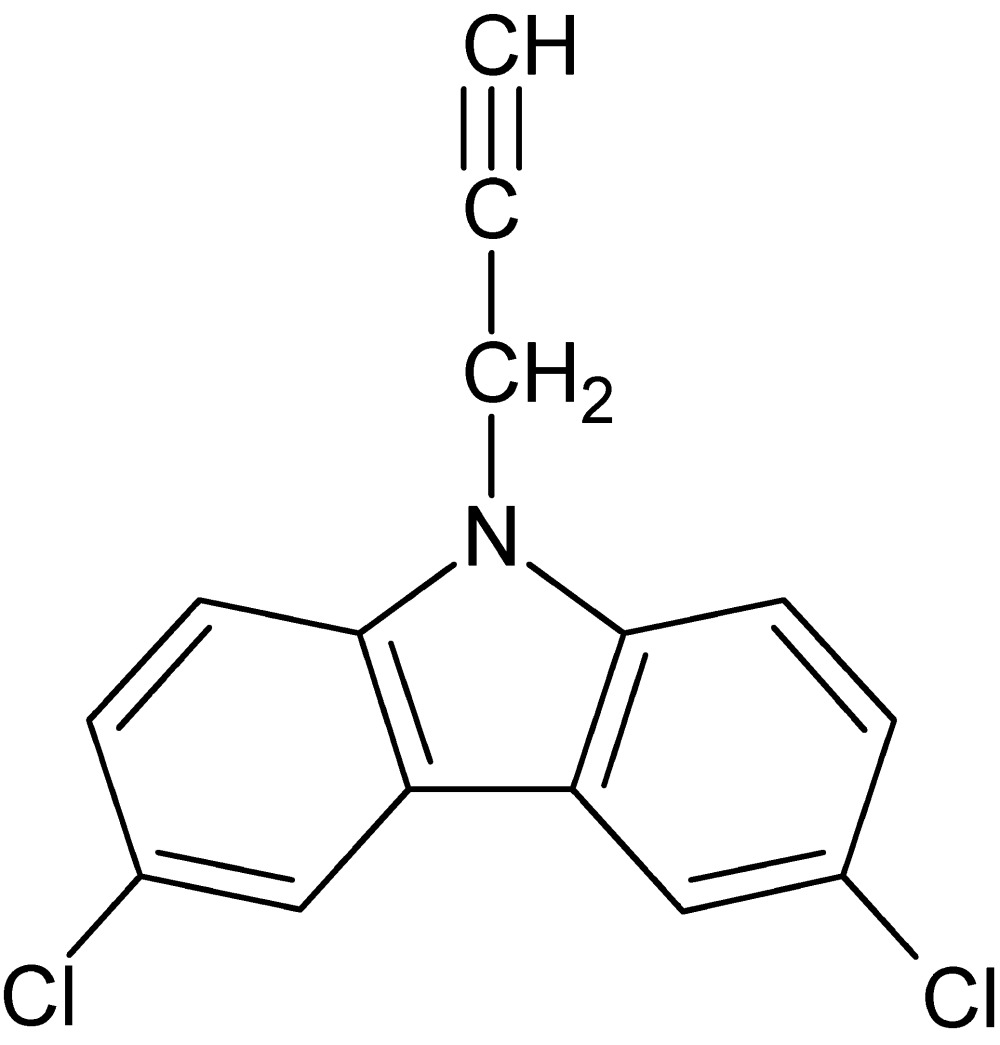


## Experimental   

### 

#### Crystal data  

C_15_H_9_Cl_2_N
*M* *_r_* = 274.13
Orthorhombic, 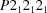

*a* = 3.9825 (1) Å
*b* = 11.1705 (4) Å
*c* = 27.4417 (9) Å
*V* = 1220.79 (7) Å^3^
*Z* = 4
Cu *K*α radiation
μ = 4.59 mm^−1^
*T* = 100 K
0.18 × 0.05 × 0.02 mm

#### Data collection  

Bruker D8 VENTURE PHOTON 100 CMOS diffractometer
Absorption correction: multi-scan (*SADABS*; Bruker, 2013 ▶) *T*_min_ = 0.74, *T*_max_ = 0.91
10712 measured reflections
2224 independent reflections
2160 reflections with *I* > 2σ(*I*)
*R*_int_ = 0.088

#### Refinement  

*R*[*F* ^2^ > 2σ(*F* ^2^)] = 0.026
*wR*(*F* ^2^) = 0.068
*S* = 1.05
2224 reflections
163 parameters
H-atom parameters constrained
Δρ_max_ = 0.22 e Å^−3^
Δρ_min_ = −0.18 e Å^−3^
Absolute structure: Flack parameter determined using 817 quotients [(*I* ^+^)−(*I* ^−^)]/[(*I* ^+^)+(*I* ^−^)] (Parsons *et al.*, 2013 ▶)
Absolute structure parameter: −0.002 (12)

### 

Data collection: *APEX2* (Bruker, 2013 ▶); cell refinement: *SAINT* (Bruker, 2013 ▶); data reduction: *SAINT*; program(s) used to solve structure: *SHELXT* (Sheldrick, 2008 ▶); program(s) used to refine structure: *SHELXL2013* (Sheldrick, 2008 ▶); molecular graphics: *ORTEP-3 for Windows* (Farrugia, 2012 ▶); software used to prepare material for publication: *WinGX* (Farrugia, 2012 ▶) and *PLATON* (Spek, 2009 ▶).

## Supplementary Material

Crystal structure: contains datablock(s) global, I. DOI: 10.1107/S1600536813032777/lh5674sup1.cif

Structure factors: contains datablock(s) I. DOI: 10.1107/S1600536813032777/lh5674Isup2.hkl

Supporting information file. DOI: 10.1107/S1600536813032777/lh5674Isup3.cml

Additional supporting information:  crystallographic information; 3D view; checkCIF report

## Figures and Tables

**Fig. 1.**
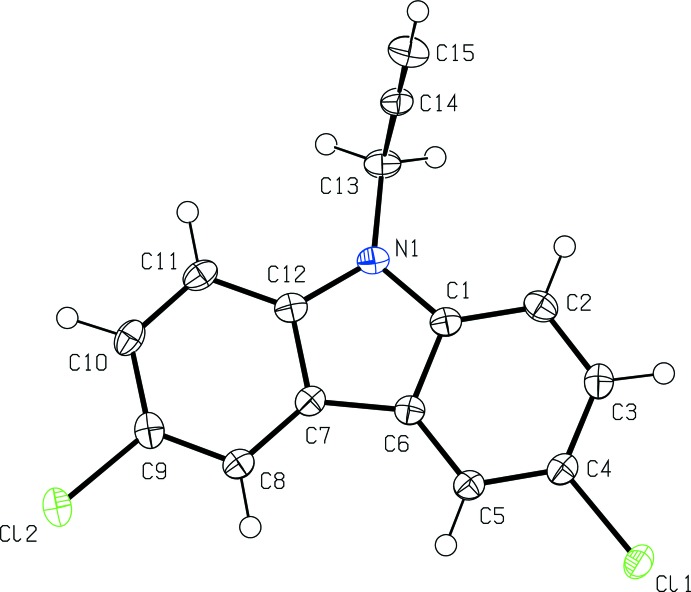
Perspective view of the title compound with 50% probability ellipsoids.

**Fig. 2.**
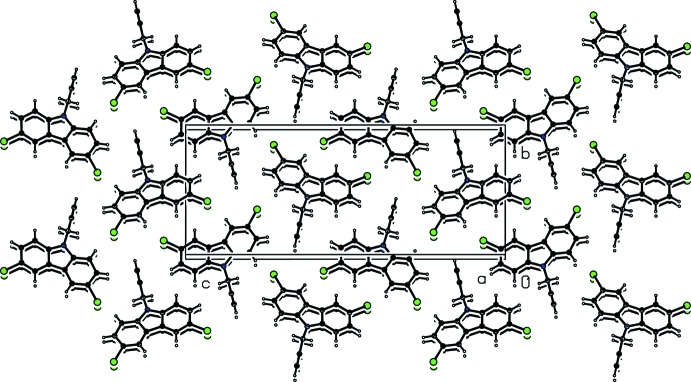
Packing of the title compound viewed along the a axis.

## supplementary crystallographic information

### 1. Comment

In recent years, carbazoles have been used as photoconductors, semiconductors and for their light-emitting properties, making them interesting organic tools for physics experiments (Zhang *et al.*, 1998). Moreover, several alkaloids based on a carbazole structure are known to possess interesting biological activities such as antitumor, antibacterial, anti-inflammatory, psychotropic and anti-histamine properties (Liu & Larock, 2007). Many synthetic carbazole derivatives are of significant pharmacological relevance because of their antifungal, antibiotic, and antitumor activities (Hussain *et al.*, 2011; Zhang *et al.*, 2010; Conchon *et al.*, 2006). The introduction of functional groups onto the carbazole scaffold is essential to generate compounds suitable for biological and physical investigations. Based on such facts we herein report the crystal structure of the title compound.

In the title compound (Fig. 1), the carbazole ring system is essentially planar (r.m.s. deviation = 0.002 Å). The Cl1 and Cl2 atoms lie slightly out of the plane of the carbazole ring system which makes angles of 1.23 (8) and 1.14 (8)° with the Cl1—C4 and Cl2—C9 bonds, respectively. The values of the geometric parameters of the title compound are within normal ranges (Xie *et al.*, 2012).

In the crystal, no classical hydrogen bonds are observed. The crystal packing is stabilized by weak van der Waals interactions. The packing of the title compound viewed along the *a* axis are shown in Fig. 2.

### 2. Experimental

Propargyl bromide (1.1 g, 9 mmol) was added to a suspension solution of 3,6-dichloro-9*H*-carbazole (0.7 g, 3 mmol) and K_2_CO_3_ (0.82 g, 6 mmol) in DMF (15 ml) and stirred at room temperature for 6 h. The excess solvent was evaporated to dryness *in vacuo*. The residue was diluted with water and then extracted with CH_2_Cl_2_ (3 *x* 30 ml). The combined organic extracts were dried over anhydrous Na_2_SO_4_, filtered and evaporated under reduced pressure to give the corresponding product as colourless crystals (0.61 g, 75%) yield, mp 469–471 K.

### 3. Refinement

All H atoms were placed geometrically and refined using a riding model with C—H = 0.95 - 0.99 Å, and with *U*_iso_(H) = 1.2*U*_iso_(C).

### Crystal data

**Table:**

C_15_H_9_Cl_2_N	*F*(000) = 560
*M**_r_* = 274.13	*D*_x_ = 1.492 Mg m^−^^3^
Orthorhombic, *P*2_1_2_1_2_1_	Cu *K*α radiation, λ = 1.54178 Å
Hall symbol: P 2ac 2ab	Cell parameters from 9151 reflections
*a* = 3.9825 (1) Å	θ = 4.3–68.0°
*b* = 11.1705 (4) Å	µ = 4.59 mm^−^^1^
*c* = 27.4417 (9) Å	*T* = 100 K
*V* = 1220.79 (7) Å^3^	Column, colourless
*Z* = 4	0.18 × 0.05 × 0.02 mm

### Data collection

**Table:**

Bruker D8 VENTURE PHOTON 100 CMOS diffractometer	2224 independent reflections
Radiation source: INCOATEC IµS micro–focus source	2160 reflections with *I* > 2σ(*I*)
Mirror monochromator	*R*_int_ = 0.088
Detector resolution: 10.4167 pixels mm^-1^	θ_max_ = 68.1°, θ_min_ = 3.2°
ω scans	*h* = −4→4
Absorption correction: multi-scan (*SADABS*; Bruker, 2013)	*k* = −13→13
*T*_min_ = 0.74, *T*_max_ = 0.91	*l* = −32→33
10712 measured reflections	

### Refinement

**Table:**

Refinement on *F*^2^	Hydrogen site location: inferred from neighbouring sites
Least-squares matrix: full	H-atom parameters constrained
*R*[*F*^2^ > 2σ(*F*^2^)] = 0.026	W = 1/[Σ^2^(*F*O^2^) + (0.0351*P*)^2^ + 0.195*P*] where *P* = (*F*O^2^ + 2*F*C^2^)/3
*wR*(*F*^2^) = 0.068	(Δ/σ)_max_ < 0.001
*S* = 1.05	Δρ_max_ = 0.22 e Å^−^^3^
2224 reflections	Δρ_min_ = −0.18 e Å^−^^3^
163 parameters	Absolute structure: Flack parameter determined using 817 quotients [(*I*^+^)-(*I*^-^)]/[(*I*^+^)+(*I*^-^)] (Parsons *et al.*, 2013)
0 restraints	Absolute structure parameter: −0.002 (12)

### Special details

**Table:**

Experimental. ^1^H-NMR (300 MHz, CDCl_3_): d 3.31 (s, 1H, C—CH), 5.34 (d, 2H, J=2.4 Hz, CH_2_—C), 7.54 (m, 2H, Ar—H), 7.74 (m, 2H, Ar—H), 8.34 (s, 2H, Ar—H). 13 C-NMR (75 MHz, CDCl_3_): d 32.2 (CH_2_), 74.8 (C—CH), 78.5 (C—CH), 111.4, 120.5, (4CH-Ar), 122.9, 124.2 (4 C-Ar), 126.4 (2CH-Ar), 138.6 (2 C-Ar). MS: (EI) m/*z* (%): 275 (*M*+2, 70), 274 (*M*+1, 74), 273 (*M*+, 100), 247 (10), 233 (44), 201 (4), 174 (2), 164 (14), 150 (2), 122 (2), 98 (2), 75 (4). Anal. for C_15_H_9_Cl_2_N: calcd. C, 65.72; H, 3.31; Cl, 25.86; N, 5.11. Found: C, 65.38; H, 3.11; N, 4.95%.
Geometry. Bond distances, angles *etc*. have been calculated using the rounded fractional coordinates. All su's are estimated from the variances of the (full) variance-covariance matrix. The cell e.s.d.'s are taken into account in the estimation of distances, angles and torsion angles
Refinement. Refinement on *F*^2^ for ALL reflections except those flagged by the user for potential systematic errors. Weighted *R*-factors *wR* and all goodnesses of fit *S* are based on *F*^2^, conventional *R*-factors *R* are based on *F*, with *F* set to zero for negative *F*^2^. The observed criterion of *F*^2^ > σ(*F*^2^) is used only for calculating -*R*-factor-obs *etc*. and is not relevant to the choice of reflections for refinement. *R*-factors based on *F*^2^ are statistically about twice as large as those based on *F*, and *R*-factors based on ALL data will be even larger.

### Fractional atomic coordinates and isotropic or equivalent isotropic displacement parameters (Å^2^)

**Table:**

	*x*	*y*	*z*	*U*_iso_*/*U*_eq_	
Cl1	0.52328 (16)	0.39290 (5)	−0.06840 (2)	0.0240 (2)	
Cl2	0.52780 (15)	0.15240 (5)	0.22392 (2)	0.0252 (2)	
N1	0.0291 (5)	0.57255 (17)	0.11884 (6)	0.0190 (5)	
C1	0.1356 (6)	0.5462 (2)	0.07181 (8)	0.0180 (6)	
C2	0.0865 (6)	0.6107 (2)	0.02874 (8)	0.0218 (7)	
C3	0.2076 (6)	0.5615 (2)	−0.01405 (8)	0.0214 (7)	
C4	0.3762 (6)	0.4513 (2)	−0.01335 (8)	0.0197 (6)	
C5	0.4309 (6)	0.3879 (2)	0.02890 (8)	0.0190 (6)	
C6	0.3072 (6)	0.4360 (2)	0.07226 (8)	0.0175 (6)	
C7	0.3067 (6)	0.3947 (2)	0.12215 (8)	0.0177 (6)	
C8	0.4327 (6)	0.2920 (2)	0.14472 (8)	0.0188 (6)	
C9	0.3751 (6)	0.2800 (2)	0.19418 (8)	0.0208 (7)	
C10	0.2040 (6)	0.3660 (2)	0.22166 (8)	0.0217 (7)	
C11	0.0803 (6)	0.4677 (2)	0.19960 (8)	0.0213 (7)	
C12	0.1317 (6)	0.4812 (2)	0.14950 (8)	0.0183 (6)	
C13	−0.1714 (6)	0.6755 (2)	0.13263 (9)	0.0219 (7)	
C14	0.0264 (6)	0.7838 (2)	0.14305 (8)	0.0216 (6)	
C15	0.1835 (7)	0.8710 (2)	0.15139 (10)	0.0284 (7)	
H2	−0.02610	0.68560	0.02880	0.0260*	
H3	0.17640	0.60250	−0.04400	0.0260*	
H5	0.54890	0.31400	0.02860	0.0230*	
H8	0.55280	0.23300	0.12690	0.0230*	
H10	0.17270	0.35420	0.25560	0.0260*	
H11	−0.03610	0.52690	0.21790	0.0260*	
H13A	−0.30450	0.65470	0.16190	0.0260*	
H13B	−0.33110	0.69340	0.10600	0.0260*	
H15	0.31000	0.94120	0.15810	0.0340*	

### Atomic displacement parameters (Å^2^)

**Table:**

	*U*^11^	*U*^22^	*U*^33^	*U*^12^	*U*^13^	*U*^23^
Cl1	0.0287 (3)	0.0266 (3)	0.0167 (2)	−0.0027 (3)	0.0020 (2)	−0.0015 (2)
Cl2	0.0256 (3)	0.0274 (3)	0.0226 (3)	−0.0015 (3)	−0.0015 (2)	0.0073 (2)
N1	0.0184 (9)	0.0176 (9)	0.0209 (9)	−0.0002 (9)	0.0008 (8)	−0.0031 (7)
C1	0.0165 (11)	0.0170 (11)	0.0206 (11)	−0.0036 (9)	−0.0012 (9)	−0.0023 (9)
C2	0.0201 (11)	0.0185 (11)	0.0269 (12)	−0.0009 (10)	−0.0024 (9)	0.0002 (9)
C3	0.0221 (11)	0.0228 (12)	0.0192 (11)	−0.0057 (10)	−0.0024 (9)	0.0016 (10)
C4	0.0191 (11)	0.0212 (11)	0.0189 (11)	−0.0052 (9)	0.0009 (9)	−0.0023 (9)
C5	0.0186 (11)	0.0180 (11)	0.0204 (10)	−0.0036 (10)	0.0004 (9)	−0.0017 (9)
C6	0.0157 (10)	0.0174 (11)	0.0195 (11)	−0.0028 (9)	−0.0017 (9)	−0.0014 (9)
C7	0.0155 (10)	0.0188 (11)	0.0188 (11)	−0.0045 (9)	0.0006 (8)	−0.0018 (9)
C8	0.0168 (11)	0.0201 (10)	0.0194 (10)	−0.0028 (9)	−0.0008 (9)	−0.0028 (9)
C9	0.0178 (12)	0.0235 (12)	0.0211 (11)	−0.0056 (10)	−0.0038 (9)	0.0031 (10)
C10	0.0194 (11)	0.0291 (13)	0.0167 (10)	−0.0066 (10)	0.0015 (9)	−0.0024 (10)
C11	0.0193 (12)	0.0238 (12)	0.0207 (11)	−0.0041 (10)	0.0017 (9)	−0.0064 (9)
C12	0.0143 (11)	0.0188 (11)	0.0219 (11)	−0.0034 (9)	−0.0008 (9)	−0.0031 (9)
C13	0.0173 (11)	0.0208 (12)	0.0276 (12)	0.0012 (10)	0.0005 (9)	−0.0043 (10)
C14	0.0212 (12)	0.0219 (11)	0.0217 (10)	0.0062 (11)	0.0028 (10)	−0.0018 (9)
C15	0.0276 (12)	0.0206 (12)	0.0369 (14)	0.0005 (11)	0.0004 (11)	−0.0031 (11)

### Geometric parameters (Å, º)

**Table:**

Cl1—C4	1.747 (2)	C9—C10	1.399 (3)
Cl2—C9	1.751 (2)	C10—C11	1.378 (3)
N1—C1	1.390 (3)	C11—C12	1.398 (3)
N1—C12	1.384 (3)	C13—C14	1.472 (3)
N1—C13	1.450 (3)	C14—C15	1.180 (3)
C1—C2	1.398 (3)	C2—H2	0.9500
C1—C6	1.408 (3)	C3—H3	0.9500
C2—C3	1.383 (3)	C5—H5	0.9500
C3—C4	1.402 (3)	C8—H8	0.9500
C4—C5	1.376 (3)	C10—H10	0.9500
C5—C6	1.395 (3)	C11—H11	0.9500
C6—C7	1.445 (3)	C13—H13A	0.9900
C7—C8	1.397 (3)	C13—H13B	0.9900
C7—C12	1.408 (3)	C15—H15	0.9500
C8—C9	1.383 (3)		
			
C1—N1—C12	108.55 (18)	N1—C12—C7	109.15 (19)
C1—N1—C13	125.32 (19)	N1—C12—C11	129.3 (2)
C12—N1—C13	126.05 (18)	C7—C12—C11	121.5 (2)
N1—C1—C2	129.3 (2)	N1—C13—C14	114.1 (2)
N1—C1—C6	108.97 (19)	C13—C14—C15	179.7 (3)
C2—C1—C6	121.7 (2)	C1—C2—H2	121.00
C1—C2—C3	117.7 (2)	C3—C2—H2	121.00
C2—C3—C4	120.3 (2)	C2—C3—H3	120.00
Cl1—C4—C3	118.46 (17)	C4—C3—H3	120.00
Cl1—C4—C5	118.91 (17)	C4—C5—H5	121.00
C3—C4—C5	122.6 (2)	C6—C5—H5	121.00
C4—C5—C6	117.7 (2)	C7—C8—H8	121.00
C1—C6—C5	120.0 (2)	C9—C8—H8	121.00
C1—C6—C7	106.67 (19)	C9—C10—H10	120.00
C5—C6—C7	133.3 (2)	C11—C10—H10	120.00
C6—C7—C8	133.0 (2)	C10—C11—H11	121.00
C6—C7—C12	106.66 (19)	C12—C11—H11	121.00
C8—C7—C12	120.3 (2)	N1—C13—H13A	109.00
C7—C8—C9	117.1 (2)	N1—C13—H13B	109.00
Cl2—C9—C8	118.59 (17)	C14—C13—H13A	109.00
Cl2—C9—C10	118.49 (17)	C14—C13—H13B	109.00
C8—C9—C10	122.9 (2)	H13A—C13—H13B	108.00
C9—C10—C11	120.2 (2)	C14—C15—H15	180.00
C10—C11—C12	117.9 (2)		
			
C12—N1—C1—C2	178.9 (2)	C3—C4—C5—C6	1.0 (4)
C12—N1—C1—C6	0.4 (3)	C4—C5—C6—C1	−0.5 (3)
C13—N1—C1—C2	1.9 (4)	C4—C5—C6—C7	177.8 (2)
C13—N1—C1—C6	−176.6 (2)	C1—C6—C7—C8	179.0 (3)
C1—N1—C12—C7	0.0 (3)	C1—C6—C7—C12	0.6 (3)
C1—N1—C12—C11	−179.3 (2)	C5—C6—C7—C8	0.6 (5)
C13—N1—C12—C7	177.0 (2)	C5—C6—C7—C12	−177.8 (3)
C13—N1—C12—C11	−2.4 (4)	C6—C7—C8—C9	−177.8 (2)
C1—N1—C13—C14	−86.6 (3)	C12—C7—C8—C9	0.4 (3)
C12—N1—C13—C14	97.0 (3)	C6—C7—C12—N1	−0.4 (3)
N1—C1—C2—C3	−177.2 (2)	C6—C7—C12—C11	179.0 (2)
C6—C1—C2—C3	1.1 (4)	C8—C7—C12—N1	−179.1 (2)
N1—C1—C6—C5	178.1 (2)	C8—C7—C12—C11	0.3 (4)
N1—C1—C6—C7	−0.6 (3)	C7—C8—C9—Cl2	179.84 (17)
C2—C1—C6—C5	−0.6 (4)	C7—C8—C9—C10	−1.0 (4)
C2—C1—C6—C7	−179.3 (2)	Cl2—C9—C10—C11	179.94 (18)
C1—C2—C3—C4	−0.7 (3)	C8—C9—C10—C11	0.8 (4)
C2—C3—C4—Cl1	179.85 (18)	C9—C10—C11—C12	0.0 (3)
C2—C3—C4—C5	−0.4 (4)	C10—C11—C12—N1	178.7 (2)
Cl1—C4—C5—C6	−179.26 (18)	C10—C11—C12—C7	−0.6 (4)
